# Association between visual emphysema and lung nodules on low-dose CT scan in a Chinese Lung Cancer Screening Program (Nelcin-B3)

**DOI:** 10.1007/s00330-022-08884-3

**Published:** 2022-06-09

**Authors:** Xiaofei Yang, Monique D. Dorrius, Wenzhen Jiang, Zhenhui Nie, Rozemarijn Vliegenthart, Harry J. M. Groen, Marjolein A. Heuvelmans, Grigory Sidorenkov, Marleen Vonder, Zhaoxiang Ye, Geertruida H. de Bock

**Affiliations:** 1 Department of Epidemiology, University Medical Center Groningen, University of Groningen, P.O. Box 30 001, FA 40, 9700 RB Groningen, The Netherlands; 2 Department of Radiology, University Medical Center Groningen, University of Groningen, Groningen, The Netherlands; 3 Department of Radiology, Tianjin Medical University Cancer Institute and Hospital, National Clinical Research Center for Cancer, Key Laboratory of Cancer Prevention and Therapy, Tianjin’s Clinical Research Center for Cancer, Huanhuxi Road, Hexi District, Tianjin, 300060 China; 4 Department of Pulmonary Diseases, University Medical Center Groningen, University of Groningen, Groningen, The Netherlands

**Keywords:** CT, General population, Pulmonary nodule, Pulmonary emphysema, Screening

## Abstract

**Objectives:**

This study aimed to evaluate the association between visual emphysema and the presence of lung nodules, and Lung-RADS category with low-dose CT (LDCT).

**Methods:**

Baseline LDCT scans of 1162 participants from a lung cancer screening study (Nelcin-B3) performed in a Chinese general population were included. The presence, subtypes, and severity of emphysema (at least trace) were visually assessed by one radiologist. The presence, size, and classification of non-calcified lung nodules (≥ 30 mm^3^) and Lung-RADS category were independently assessed by another two radiologists. Multivariable logistic regression and stratified analyses were performed to estimate the association between emphysema and lung nodules, Lung-RADS category, after adjusting for age, sex, BMI, smoking status, pack-years, and passive smoking.

**Results:**

Emphysema and lung nodules were observed in 674 (58.0%) and 424 (36.5%) participants, respectively. Participants with emphysema had a 71% increased risk of having lung nodules (adjusted odds ratios, aOR: 1.71, 95% CI: 1.26–2.31) and 70% increased risk of positive Lung-RADS category (aOR: 1.70, 95% CI: 1.09–2.66) than those without emphysema. Participants with paraseptal emphysema (*n* = 47, 4.0%) were at a higher risk for lung nodules than those with centrilobular emphysema (CLE) (aOR: 2.43, 95% CI: 1.32–4.50 and aOR: 1.60, 95% CI: 1.23–2.09, respectively). Only CLE was associated with positive Lung-RADS category (*p *= 0.02). CLE severity was related to a higher risk of lung nodules (ranges aOR: 1.44–2.61, overall *p* < 0.01).

**Conclusion:**

In a Chinese general population, visual emphysema based on LDCT is independently related to the presence of lung nodules (≥ 30 mm^3^) and specifically CLE subtype is related to positive Lung-RADS category. The risk of lung nodules increases with CLE severity.

**Key Points:**

*• Participants with emphysema had an increased risk of having lung nodules, especially smokers.*

*• Participants with PSE were at a higher risk for lung nodules than those with CLE, but nodules in participants with CLE had a higher risk of positive Lung-RADS category.*

*• The risk of lung nodules increases with CLE severity.*

**Supplementary Information:**

The online version contains supplementary material available at 10.1007/s00330-022-08884-3.

## Introduction

Lung cancer is the primary cause of cancer death worldwide [1]. It ranked sixth among the top 10 leading causes of death in 2019, rising from 1.2 million to 1.8 million cases since 2000. In China, which is currently the most populous country in the world, lung cancer is the malignancy with the highest incidence and the main cause of cancer-related mortality [2]. Even though the treatment of lung cancer is gradually improving, the 5-year survival rate was still as low as 16.5% in 2012–2015 [3]. The increasing lung cancer burden in China combined with poor prognosis is a challenge for cancer prevention. LDCT screening among high-risk individuals could lead to a 20–33% lung cancer-specific mortality reduction [4]. As a consequence, LDCT screening has been introduced and is now recommended as a strategy for the early detection of lung cancer worldwide [5].

With the progress of higher spatial resolution of CT scanners combined with more advanced postprocessing software, the prevalence of lung nodules in lung cancer screening may vary from 21 to 86% depending on acquisition protocol, the population included, and guidelines used during 2006–2018 [6]. Although the majority of detected lung nodules are benign, lung cancer rate in participants with non-calcified pulmonary baseline nodules ranges from 2 to 11% [7, 8]. About 3–4% of the participants with a non-calcified nodule at baseline will develop lung cancer within the following 2–5 years [9]. The risk factors for lung cancer such as size, age, and smoking have been established [10], but far less is known about risk factors for the presence of lung nodules and its malignant risk. This may be critical because lung nodules are the early manifestations of lung cancer.

Emphysema is a pathologic condition with enlargement of air spaces in the terminal bronchioles. It is one of the predictive risk factors for neoplastic transformation of the lining epithelium [11]. There are numerous studies on risk factors involving emphysema as a risk factor for lung cancer [12, 13]. Some studies on lung cancer screening suggest that 48–58% of the participants with emphysema also have lung nodules [14], which could lead to a higher lung cancer mortality because of increased susceptibility to biological damage [15]. Although emphysema and the presence of lung nodules have shared risk factors, such as age and smoking, it is not clear whether emphysema is independently associated with the presence of lung nodules. In previous studies, this could not be investigated since most studies only included a high-risk (smoking) population. However, identifying new risk factors for lung nodules or the malignant risk is important in the future to optimize nodule management of incidentally detected lung nodules in the general population.

For that, the present study aimed to evaluate the association between the presence, subtypes, and severity of visual emphysema, and the presence of lung nodules, as assessed by LDCT in a general Chinese population. The association between emphysema and malignant risk (Lung-RADS category) of lung nodules was also evaluated.

## Materials and methods

### Participants

**Fig. 1:**
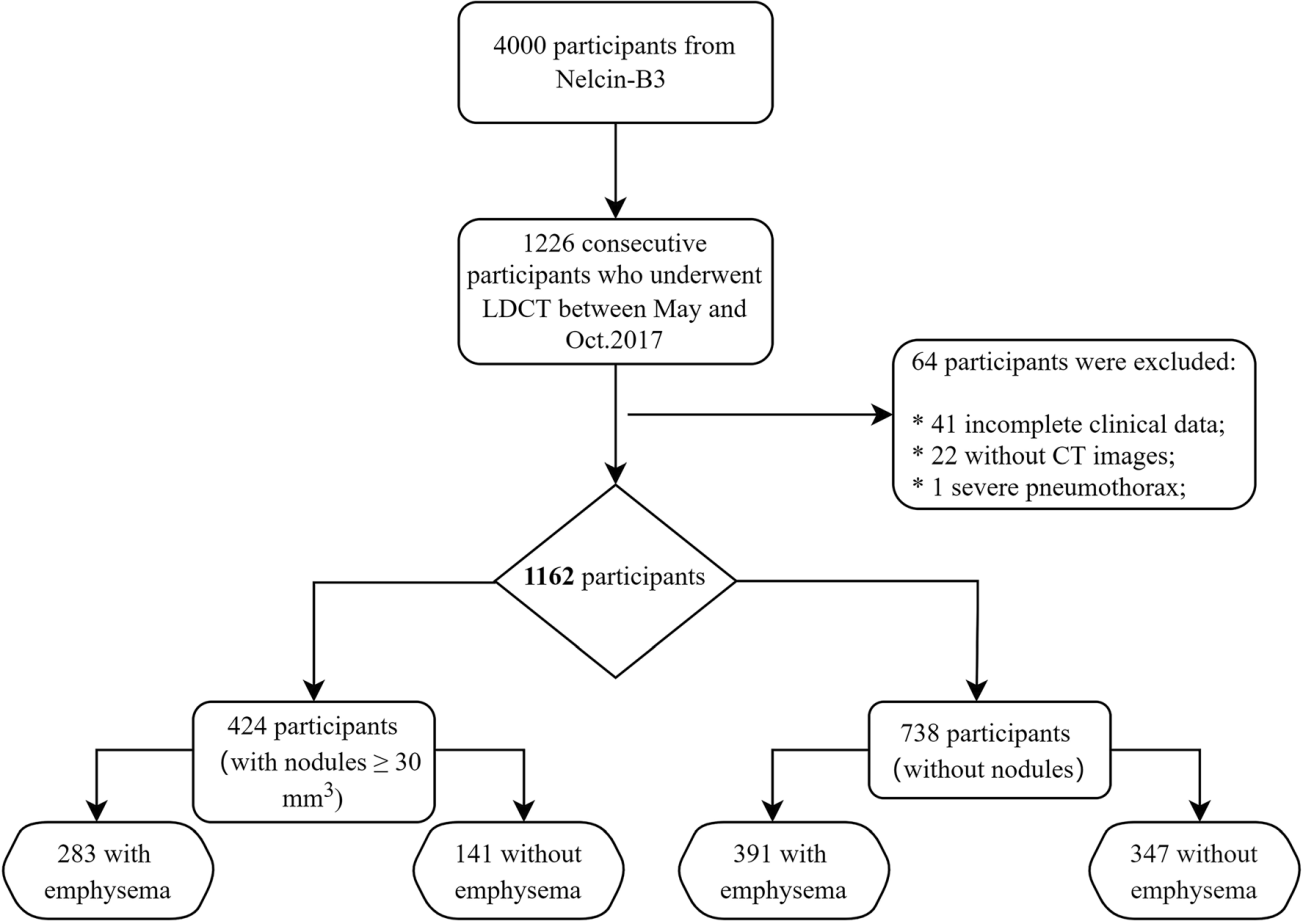
Flowchart of study design

The here presented study used data from participants that were included in the Nelcin-B3 study. In the Nelcin-B3 study, a general Chinese population was recruited without inclusion criteria on smoking and pack-years to identify risk factors for the “Big 3” diseases (lung cancer, cardiovascular disease, and COPD), and reference values based on LDCT [16]. The study was approved by the Ethics Committee of Biomedicine Research of Second Military Medical University (registration number: NCT03992833). At the Radiology Department of Tianjin Medical University Cancer Institute and Hospital (TJMUCIH), 4000 participants from the general population, being Tianjin residents for 3 years or longer 3 years, aged between 40 and 74 years, without any history of cancer, were invited for LDCT lung cancer screening. The current analysis focused on a consecutive series of the participants included in the Nelcin-B3 study that underwent LDCT between May and October 2017 (see Fig. 1). Participants were excluded if they had incomplete data or pneumothorax. All participants signed the informed consent, and a structured face-to-face interview was conducted by trained interviewers to gather demographic information (age, sex, ethnicity, smoking status, pack-years, passive smoking, BMI). Passive smoking was defined as an indoor environment where participants inhale smoke produced by others ≥ 1 day a week for ≥ 15 min. About the pack-years, zero was scored for never smoker.

### CT scan acquisition and interpretation

The CT chest examinations were performed with a 64-detector row CT system (Somaton Definition AS 64, Siemens) using a low-dose technique without the use of a contrast agent. Details of the technical parameters used in the CT scan protocol were as follows: 120 kVp, 35 mAs, pitch of 1.0, reconstruction kernels D45F (emphysema assessment), and B80F (lung nodules assessment) were applied to reconstruct images at 1.0/0.7 mm thickness and increment. All the participants were scanned in the supine position with head forward. The CT scans were obtained at deep inspiration with the breath-holding of subjects under the above technical setting.

### Lung nodule assessment

After prospective collection of LDCT scans, the detection and features of suspected lung nodules were retrospectively assessed by one junior chest radiologist (Y.F.M. with 4 years of experience) and checked by another senior chest radiologist (D.M. with 10 years of experience). The maximum intensity projection (MIP) technique was used to detect lung nodules under the D45F kernel with 10-mm slice thickness. Three-dimensional segmentation software (MM Oncology Syngo.via., version VB30A, Siemens) was used to automatically measure the volume of each detected nodule under the B80F kernel. All CT scans used for lung nodule detection were read at both lung window (window center: −500 HU, window width: 1200 HU) and mediastinal window (window center: 35 HU, window width: 320 HU). Nodules were scored and classified as solid, part-solid, and non-solid nodules (pure ground-glass nodules). Nodule location was classified as upper lobe (right middle, left, or right upper lobe) or lower lobe (left or right lower lobe). Included in the here presented analysis were all non-calcified nodules with volume ≥ 30 mm^3^ [16–18]. LDCT scan findings were assessed using Lung-RADS category (1, 2, 3, and 4) [19].

### Visual emphysema assessment

The visual emphysema assessment was performed using the Minimum Intensity Projection (Minip) technique with 10-mm thickness in the lung window setting (window center: −850 HU, window width: 400 HU) [20] and Multiplanar Reconstruction (MPR) technique with 1-mm thickness in window setting (window center: −750 HU, window width: 700 HU) [21] all based on the D45F reconstruction kernel CT images with the same software used for nodule assessment. All images were visually assessed by one radiologist (X.F.Y. with 5 years of experience) using a standard protocol based on validated criteria created by the Fleischner Society [22]. Interobserver agreement was determined based on 100 randomly selected cases assessed by a second radiologist (Z.H.Y. with 2 years of experience).

Emphysema was scored as well-defined or ill-defined low attenuation or lucencies following the Fleischner criteria [22]. If present (at least trace), emphysema was further categorized as one of the three predominant subtypes of emphysema (centrilobular[CLE], paraseptal [PSE], and panlobular). The severity of CLE was categorized into trace (< 0.5%), mild (0.5–5%), moderate (> 5%), confluent and advanced destructive (confluent-ADE) according to the morphology and most severe percentage of lucency in a lung zone.

### Statistical methods

Kappa statistics for the presence of emphysema and weighted kappa coefficients for CLE and PSE severity were calculated to assess interobserver agreement. Participants were classified as having no nodule versus having at least one nodule, and negative Lung-RADS (1 or 2) versus positive (3 or 4). Baseline characteristics of participants were described, overall and stratified by the presence or absence of lung nodules. To estimate the association between the baseline characteristics and the presence of lung nodules, univariate logistic regression analysis was performed to estimate odds ratios (ORs) and related 95% confidence intervals (95% CIs). To adjust for covariates, the following characteristics were selected: age, sex, smoking status, pack-years, passive smoking, and BMI. Those were based on previous literature [23, 24]. Multivariable logistic regression analysis was performed with “enter approach” to estimate aORs and related 95% CIs. In addition, an interaction term for smoking status and emphysema was included in the model. Given that analysis was significant, a stratified analysis was performed for smokers (current smokers) and non-smokers (never or former smokers). The predictors of interest were as follows: the presence of emphysema, the subtypes of emphysema, and the severity of CLE. The severity of emphysema was entered as an ordinal variable to investigate the effect in each category and as a numerical variable to test for linear trend. Mann-Whitney *U* testing and chi-square testing were conducted to analyze the association between emphysema and size, number, classification, and location of lung nodules. The nodule size, classification, and location were determined according to the largest nodule, and the highest Lung-RADS category was taken into account when more than one nodule was present. The number of nodules was categorized into three categories: 1 nodule, 2–3 nodules, 4 and more nodules. Statistical analysis was conducted using the SPSS 23.0 (IBM Corporation). *p* < 0.05 was considered a statistically significant difference.

## Results

### Participant characteristics

In total, 1162 participants were included in this analysis (see Fig. 1). The mean age of the participants was 61.2 ± 6.9 years, 517 (44.5%) were male, and 781 (67.2%) were never smokers (see Table 1). Of the 1162 participants, 424 (36.5%) had lung nodules and 674 (58.0%) participants had emphysema. Concerning the predominant subtypes of emphysema, the most frequent emphysema subtype was centrilobular (93%) including 58 (9.3% ) moderate, confluent, or ADE severity, followed by PSE (7.0%). There were no participants with panlobular emphysema.

**Table 1:** Characteristics of participants, overall and stratified by participants with and without any lung nodules (*n*=1162)

Variables	Overall	With lung nodules (*n* = 424)	Without any lung nodules (*n* = 738)	cOR	95% CI	*p* value
Age Mean ± SD	61.2 ± 6.9	61.8 ± 6.6	60.8 ± 7.1	1.02	1.00–1.04	**0.03**
Ethnicity						
Minority	14 (1.2%)	5 (1.2%)	9 (1.2%)	1		
Han	1148 (98.8%)	419 (98.8%)	729 (98.8%)	1.04	0.34–3.11	0.95
Sex						
Female	645 (55.5%)	210 (49.5%)	435 (58.9%)	1		
Male	517 (44.5%)	214 (50.5%)	303 (41.1%)	1.46	1.15–1.86	**< 0.01**
Smoking status						0.15
Never	781 (67.2%)	272 (64.2%)	509 (69.0%)	1		
Former	110 (9.5%)	44 (10.4%)	66 (8.9%)	1.25	0.83–1.88	0.29
Current	271 (23.3%)	108 (25.5%)	163 (22.1%)	1.24	0.93–1.65	0.14
Passive Smoking						
No (< 10 years)	681 (58.6%)	247 (58.3%)	434 (58.8%)	1		
Yes (≥ 10 years)	481 (41.4%)	177 (41.7%)	304 (41.2%)	1.02	0.80–1.30	0.85
Pack-years						
< 10	859 (73.9%)	304 (71.7%)	555 (75.2%)	1		
≥ 10	303 (26.1%)	120 (28.3%)	183 (24.8%)	1.20	0.91–1.57	0.19
BMI (kg/m^2^)						
< 25	657 (56.5%)	246 (58.0%)	411 (55.7%)	1		
≥ 25	505 (43.5%)	178 (42.0%)	327 (44.3%)	0.91	0.71–1.16	0.44
Emphysema						
No	488 (42.0%)	141 (33.3%)	347 (47.0%)	1		
Yes	674 (58.0%)	283 (66.7%)	391 (53.0%)	1.78	1.39–2.28	**< 0.001**
Predominant subtypes						**< 0.01**
None	488 (42.0%)	141 (33.3%)	347(47.0%)	1		
CLE	627 (54.0%)	259 (61.3%)	368 (49.9%)	1.73	1.35–2.24	**< 0.001**
PSE	47 (4.0%)	24 (5.7%)	23 (3.1%)	2.57	1.40–4.70	**< 0.01**
Severity of CLE						**< 0.01**
None	488 (43.8%)	141 (35.3%)	347 (48.5%)	1		
Trace	438 (39.3%)	166 (41.5%)	272 (38.0%)	1.50	1.14–2.00	**< 0.01**
Mild	131 (11.7%)	62 (15.5%)	69 (9.7%)	2.21	1.49–3.28	**< 0.001**
Moderate-ADE	58 (5.2%)	31 (7.8%)	27 (3.8%)	2.83	1.63–4.91	**< 0.001**

Note: Abbreviation: *cOR* crude odds ratios, *BMI* body mass index, *CLE* centrilobular emphysema, *PSE* paraseptal emphysema, *Moderate-ADE* moderate, confluent or advanced destructive

Significant *p* values are marked in bold

### Interobserver agreement

Agreement between radiologist for assessment of presence of emphysema was good (*κ* = 0.76 (95% CI: 0.63–0.89). Similarly, agreement was good for severity of CLE (*κ*_weighted_ = 0.77, 95% CI: 0.67–0.88) and PSE (*κ*_weighted_=0.77, 95% CI: 0.58–0.96).

### Association between participant characteristics and lung nodules presence

Participants with lung nodules were slightly older (mean 61.8 vs 60.8 years, OR: 1.02, 95% CI: 1.00–1.04), and were more frequently male (50.5% vs 49.5%, OR: 1.46, 95% CI: 1.15–1.86), compared to participants without lung nodules (see Table 1). Furthermore, participants with lung nodules had a higher prevalence of visual emphysema (66.7% vs 53.0%, OR: 1.78, 95% CI: 1.39–2.28). There were no significant differences regarding ethnicity, smoking status, pack-years, BMI, and passive smoking between participants with and without lung nodules.

### Association between emphysema and lung nodules presence, Lung-RADS category

Multivariable analysis showed that participants with emphysema based on visual assessment increased the risk of lung nodules by 71% (aOR: 1.71, 95% CI: 1.26–2.31; see Table 2) compared to participants without emphysema. Stratified results showed that the higher risk in participants with emphysema was more pronounced in smokers (aOR: 2.28, 95% CI: 1.17–4.43), but was also seen in non-smokers (aOR: 1.55, 95% CI: 1.16–2.06). PSE seemed to be associated with a somewhat higher risk of lung nodules than CLE (aOR: 2.43, 95% CI: 1.32–4.50, and aOR: 1.60, 95% CI: 1.23–2.09, respectively), compared with participants without any emphysema (see Table 3). With respect to emphysema severity, the aORs for lung nodules gradually increased (aOR range: 1.44–2.61, overall *p* < 0.01, see Table 4) with the severity of CLE. The aOR for lung nodule was still significantly different when emphysema severity was considered a continuous variable (aOR: 1.40, 95% CI: 1.19–1.64, *p* for trend < 0.001).

**Table 2:** Multivariable associations between the presence of emphysema and the presence of lung nodules, stratified by smoking status

Variables	Overall (1162)		Smokers (*n* = 271)		Non-smokers (*n* = 891)	
	aOR (95% CI)	*p* value	aOR (95% CI)	*p* value	aOR (95% CI)	*p* value
Emphysema						
No	1		1			
Yes	1.71 (1.26–2.31)	**< 0.01**	2.28 (1.17–4.43)	0.02	1.55 (1.16–2.06)	**< 0.01**
Age	1.01 (0.99–1.03)	0.38	1.02 (0.98–1.07)	0.28	1.00 (0.98–1.03)	0.71
Sex						
Female	1		1			
Male	1.44 (1.02–2.05)	**0.04**	1.82 (0.56–5.94)	0.32	1.41 (1.00–1.99)	**0.04**
Smoking status *emphysema		**0.04**				
Smoking status						
Never	1		--	--	--	--
Former	1.56 (0.70–3.50)	0.28	--	--	--	--
Current	0.60 (0.30–1.19)	0.14	--	--	--	--
Pack-years	1.00 (0.99–1.02)	0.61	1.01 (0.99–1.02)	0.46	0.99 (0.98–1.01)	0.50
Passive smoking (years)						
No (< 10 )	1		1		1	
Yes (≥ 10)	0.98 (0.76–1.27)	0.87	0.65 (0.38–1.10)	0.11	1.12 (0.84–1.51)	0.44
BMI (kg/m^2^)						
< 25	1	1	1		1	
≥ 25	0.94 (0.74–1.21)	0.64	1.00 (0.59–1.69)	0.99	0.93 (0.79–1.23)	0.62

Note: Abbreviation: *aOR* adjusted odds ratios, *BMI* body mass index. Adjusted for: age, sex, smoking status, pack-years, passive smoking, and BMI; *interaction between smoking status and emphysema

Significant *p* values are marked in bold

**Table 3:** Multivariable associations between subtypes of emphysema and the presence of lung nodules

Variables	Multivariable analysis		
	aOR	95% CI	*p* value
Predominant subtypes			**< 0.01**
No	1		
CLE	1.60	1.23–2.09	**< 0.01**
PSE	2.43	1.32–4.50	**< 0.01**

Note: Abbreviation: *aOR* adjusted odds ratios, *CLE* centrilobular emphysema, *PSE* paraseptal emphysema. Adjusted for: age, sex, smoking status, pack-years, passive smoking, and BMI

Significant *p* values are marked in bold

**Table 4:** Multivariable associations between severity of CLE with the presence of lung nodules

Variables	Multivariable analysis					
	aOR*	95% CI	*p* value	aOR**	95% CI	*p* (for trend)
Severity of CLE			**< 0.01**			
None	1					
Trace	1.44	1.09–1.91	**0.01**	1.40	1.19–1.64	**< 0.01**
Mild	2.02	1.33–3.07	**< 0.01**			
Moderate-ADE	2.61	1.44–4.72	**< 0.01**			

Note: Abbreviation: *aOR* adjusted odds ratios, *Moderate-ADE* moderate, confluent or advanced destructive. Adjusted for: age, sex, smoking status, pack-years, passive smoking, and BMI. *Severity was analyzed as an ordinal variable, **as a numerical variable

Significant *p* values are marked in bold

The multivariable analysis also showed that presence of emphysema in a participant increased the risk of positive Lung-RADS category by 70% (aOR: 1.70, 95% CI: 1.09–2.66; see Table S1) compared to a participant without emphysema. When stratified by subtype of emphysema, CLE was associated with positive Lung-RADS category (aOR: 1.69, 95% CI: 1.08–2.66), whereas this was not shown for PSE (aOR: 1.83, 95% CI: 0.71–4.72).

### Emphysema in relation to lung nodule characteristics

Of the 424 participants with lung nodules, 284 (67.0%, 284 of 424) had a single nodule. There was a difference in the total number of nodules between those with and without emphysema (overall *p* < 0.01, see Table 5). Participants with emphysema were more likely to have 2–3 nodules versus those without (31.8% vs 17.0%, respectively) (*p* < 0.01). Participants with emphysema had larger nodules (median 74 vs 62 mm^3^, *p* = 0.03) when compared to participants without. Most participants with nodules had solid nodules (90.8%, 385 of 424), followed by non-solid nodules (6.6%, 28 of 424) and part-solid nodules (2.6%, 11 of 424). This remained true when comparing participants with and without emphysema. In 223 (52.6%, 223 of 424) participants, nodules were located in the upper lobe. No significant difference was found when comparing participants with and without emphysema.

**Table 5:** Characteristics of nodules, overall and stratified by with and without emphysema (*n* = 424)

Variables	Overall (*n* = 424)	With emphysema (*n* = 283)	Without emphysema (*n* = 141)	*p* value
Nodule volume (mm^3^) Median (Q25, Q75)	69 (43, 143)	74 (45, 152)	62 (41, 112)	0.03*
Nodule number				< 0.01**
1	283 (66.7%)	174 (61.5%)	109 (77.3%)^#^	
2–3	115 (27.1%)	91 (32.2%)	24 (17.0%)^#^	
≥ 4	26 (6.1%)	18 (6.4%)	8 (5.7%)	
Nodule classification				0.47**
Solid	385 (90.8%)	254 (89.8%)	131 (92.9%)	
Part solid	11 (2.6%)	9 (3.2%)	2 (1.4%)	
Non-solid	28 (6.6%)	20 (7.1%)	8 (5.7%)	
Nodule location				0.70**
Upper lobe	223 (52.6%)	147 (51.9%)	76 (53.9%)	
Lower lobe	201 (47.4%)	136 (48.1%)	65 (46.1%)	

Note: *Based on Mann-Whitney *U* testing; **based on chi-square testing. ^#^*p* < 0.01 vs. with emphysema; if multiple nodules were detected, the largest one was shown

Significant *p* values are marked in bold

## Discussion

In this LDCT screening study in a general Chinese population, we explored the association between the presence of emphysema and the presence of lung nodules. Participants with visual emphysema had a 71% increased risk of having at least one non-calcified lung nodule compared to those without emphysema. In particular, participants with PSE had a higher risk to have lung nodules but nodules in participants with CLE had a higher risk of positive Lung-RADS category (3 or 4). There was a severity-dependent effect; more CLE conferred a higher risk for lung nodules.

In our study, 36.5% of participants had at least one non-calcified lung nodule. This prevalence was between the result of the Dutch-Belgian lung cancer screening trial (Nelson), where 51% of participants had non-calcified nodules [25] and 27.3% reported by the National lung screening study (NLST) [26]. The main explanations for this difference are the differences in nodule detection criteria between the studies, with detection thresholds of 15 mm^3^ (Nelson), 30 mm^3^ (current study), and 4 mm (roughly 34 mm^3^, NLST). However, we would expect a lower prevalence of lung nodules in our study, as in our study younger participants without a limit of pack-years were included, while the Nelson and NLST studies included participants, aged 50 to 74 years, who had a history of at least 15 or 30 pack-years of smoking [26, 27].

In total, 58% of participants had emphysema, which is almost similar to 61% as reported in the COPDGene Study using the American population [28]. This is remarkable, since the participants in those studies had a longer cumulative tobacco exposure of 42 pack-years in current and former smokers, while in our population, participants had a mean of 8 pack-years and 67% of them were never-smokers. It is worth noting that 65% of participants with emphysema only had trace emphysema. The much more severe outdoor air pollution and indoor cooking fume in China (Beijing-Tianjin-Hebei region) compared to western countries [29, 30] may play an important role in emphysema formation since there is evidence showing outdoor air pollution and indoor cooking fume contribute to higher incidence and prevalence of emphysema [31].

We found that participants with emphysema had more than one-and-a-half-fold risk for the presence of lung nodules compared to participants without emphysema, which is somewhat higher than a previous study in which a non-significant risk was reported (OR: 1.18, 95% CI: 0.74–1.73) [14]. In our study, participants with PSE seemed to have a higher risk of lung nodules than CLE, although 95% CI overlapped. Compared with CLE, PSE is more frequently associated with marked thickening walls of bronchi with distinct airway inflammatory [22], which facilitates lung nodule formation [32]. We also observed that greater severity of CLE conferred a greater risk of lung nodules. It could be explained by the effects of smoking intensity, varied air pollution exposure, and advancing age. In the current study, regarding the malignant risk of lung cancer, participants with emphysema in our study had a 70% increased risk for positive Lung-RADS category (3 or 4). This is consistent with findings from Burnett-Hartman et al that COPD is positively associated with Lung-RADS 4 (OR, 1.78; 95% CI, 1.45–2.20) [33]. More specifically, the association found in our study was only held for participants with CLE, not PSE. Similarly, a study by Gonzalez et al showed that CLE is associated with increased lung cancer risk [34]. When we focused on the participants with lung nodules, the presence of emphysema was related to larger nodules and participants with 2–3 nodules. This is consistent with findings of a study by Ewa et al, who found that emphysema is more frequently associated with larger and multiple lung nodules [11].

Several mechanisms can explain the association between emphysema and lung nodules. Most of the nodules are not malignant in general [35]. First, local accumulations of inflammatory cells such as lymphocytes, macrophages, and neutrophils occur on external stimulus, which could facilitate lung injury resulting in emphysema and lung nodule formation [36]. Second, as the shared risk factor for emphysema and lung nodule, smoking exposure could induce oxidative stress which will cause reactive oxygen production and antioxidant reduction. The progress of oxidative stress enhances inflammation, DNA damage, and accelerated aging, which can result in emphysema, lung nodules, and ultimately lung cancer [37]. Third, air pollutants, such as nitrogen oxides and particulate matter 2.5 (PM_2.5_), are highly reactive oxidants and could cause long-term inflammation [38]. In addition, PM_2.5_ could also induce changes in long-noncoding RNAs through reactive oxygen species, thereby promoting autophagy and proliferation of lung cells, further leading to the occurrence of emphysema and lung nodules [39]. Finally, the main pathological feature of emphysema is the permanent enlargement of airspaces distal to the terminal bronchioles, which will be the risk factor for neoplastic transformation [11].

## Strengths and limitations

This study is, to the best of our knowledge, the first to analyze the association between the subtypes and severity of emphysema and the presence of lung nodules based on LDCT chest CT in a general Chinese population. Moreover, the participants in this study came from a general population with no inclusion criteria regarding the smoking status and pack-years, making the results innovative and more generalizable compared to hospital-based and other population-based screening studies. Additionally, our study had a good interobserver agreement of emphysema, which is comparable to that of study using the Fleischner Society classification system (*k* = 0.82, 95%: 0.80–0.84) [28]. This study also has some limitations. First, the number of participants with PSE and confluent-ADE of CLE was relatively small, so we merged it with moderate severity and did not perform an analysis for PSE severity. Second, this was a cross-sectional study, which limits the ability to explore the etiological relationship between emphysema and lung nodules. Third, lung function was not measured in the current study, while this might be an important confounding factor that could compromise the OR of emphysema for lung nodules after adjusting. Fourth, there is a lack of complete follow-up for lung cancer diagnosis of all participants. To overcome this limitation, the Lung-RADS risk for malignancy of nodules was added.

## Conclusion

This study shows that the presence, subtypes, and severity of emphysema are related to the presence of lung nodules in a general Chinese population. The risk for the presence of lung nodules increases with CLE severity. The presence of emphysema and specifically CLE subtype are related to the positive Lung-RADS category. The significance of these findings for lung cancer screening should be evaluated.

## Supplementary Information

ESM 1 (DOCX 18.2 kb)

## Abbreviations

aOR: Adjusted odds ratios
BMI: Body mass index
CLE: Centrilobular emphysema
cOR: Crude odds ratios
LDCT: Low-dose computed tomography
Moderate-ADE: Moderate, confluent or advanced destructive
PSE: Paraseptal emphysema

## Declarations

### Guarantor

The scientific guarantor of this publication is Prof. Geertruida H. de Bock.

### Statistics and biometry

One of the authors has significant statistical expertise.

### Methodology

• retrospective

• cross sectional study

• performed at one institution
