# Supplementary material for: Association between visual emphysema and lung nodules on low-dose CT scan in a Chinese Lung Cancer Screening Program (Nelcin-B3)
Source: Eur Radiol. 2022 Jun 9;32(12):8162–70. doi: 10.1007/s00330-022-08884-3 (PMC9705457; doi:10.1007/s00330-022-08884-3)
Supplement: Supplementary file 1 — (DOCX 18.2 kb) [file 330_2022_8884_MOESM1_ESM.docx]

**Supplement Material**

**Table S1: Multivariable associations between the presence of emphysema and Lung-RADS category, stratified by subtype of emphysema**

|  | **Lung-RADS Category** | | | **Multivariable analysis** | | | |
| --- | --- | --- | --- | --- | --- | --- | --- |
| Variables | | Positive (3 and 4)  (n=113) | Negative (1or 2) (n=1049) | | aOR | 95% CI | P value |
| Emphysema § | |  |  | |  |  |  |
| No | | 32 (28.3%) | 456 (43.5%) | | 1 |  |  |
| Yes | | 81 (71.7%) | 593 (56.5%) | | 1.70 | 1.09-2.66 | **0.02** |
| Predominant Subtypes § | |  |  | |  |  | 0.07 |
| No | | 32 (28.3%) | 456 (43.5%) | | 1 |  |  |
| CLE | | 75 (66.4%) | 552 (52.6%) | | 1.69 | 1.08-2.66 | **0.02** |
| PSE | | 6 (5.3%) | 41 (3.9%) | | 1.83 | 0.71-4.72 | 0.21 |

Note: Abbreviation: aOR, adjusted odds ratios; CLE, centrilobular emphysema; PSE, paraseptal emphysema. § Adjusted for: Age, sex, smoking status, pack-years, passive smoking, and BMI.
